# The Pre-Catching Sperm Technique Increases the Efficiency of the Intracytoplasmic Sperm Injection Method by Improving Fertilization and Blastocyst Formation Rates

**DOI:** 10.3390/jcm14144872

**Published:** 2025-07-09

**Authors:** Catherine Haering, Evelina Manvelyan, Kathryn Coyne, Lauren Alexis Hyams, James Hamrick, Joseph Findley, Rachel Weinerman, Rebecca Flyckt, Sung Tae Kim

**Affiliations:** 1 Division of Reproductive Endocrinology and Infertility, Case Western Reserve University School of Medicine, Cleveland, OH 44106, USA; cph43@case.edu (C.H.); evelina.manvelyan@uhhospitals.org (E.M.); kathryn.coyne@uhhospitals.org (K.C.); joseph.findley@uhhospitals.org (J.F.); rachel.weinerman@uhhospitals.org (R.W.); rebecca.flyckt2@uhhospitals.org (R.F.); 2 Department of Reproductive Endocrinology and Infertility, University Hospitals Ahuja, Beachwood, OH 44122, USA; lauren.hyams@uhhospitals.org (L.A.H.); james.hamrick@uhhospitals.org (J.H.)

**Keywords:** assisted reproductive technology, intracytoplasmic sperm injection, infertility, reproductive medicine

## Abstract

A retrospective cohort study was conducted comparing conventional intracytoplasmic sperm injection (ICSI) with a pre-catching sperm (PCS)-ICSI technique. Cases with at least 0.5 million motile sperm and 5 mature oocytes were included. Conventional ICSI involves simultaneous loading of sperm and oocytes onto the dish, followed by identification, immobilization, and loading of sperm into pipettes for oocyte injection. In the PCS-ICSI technique, suitable sperm were identified and immobilized prior to oocyte loading onto the dish, thus reducing the oocyte exposure time. Variables of interest included rate of fertilized and degenerated oocytes, abnormal fertilization, quality blastocyst formation, and pregnancy outcomes. Statistical analysis utilized Student’s *t*-test and Fisher’s Exact Test. Our study included 330 PCS-ICSI and 287 conventional ICSI cases. Female age, BMI, AMH, total number of collected oocytes, and rate of abnormal fertilization were similar between groups. The PCS-ICSI group demonstrated an increased rate of oocyte fertilization (84.0% vs. 79.3%, *p* < 0.001), good quality blastocyst formation (54.9% vs. 48.0%, *p* < 0.001), and a lower rate of oocyte degeneration (1.4% vs. 3.5%, *p* < 0.001). Positive pregnancy rate, clinical pregnancy, and live birth rate were similar between groups. The expansion of this technique resulted in increased oocyte fertilization and good quality blastocyst formation, and decreased oocyte degeneration. Further studies will evaluate the effectiveness of this technique in broader patient populations.

## 1. Introduction

Intracytoplasmic sperm injection (ICSI) stands as a pivotal method in assisted reproductive technology, revolutionizing the prospects of conception for couples facing infertility challenges. Successful clinical outcomes were first recorded in the early 1990s, and ICSI has only grown in popularity as an option for assisted reproductive technology (ART) [1]. Advantages of ICSI over other methods such as passive IVF or intrauterine insemination include avoidance of polyspermy, ability to fertilize multiple oocytes in one controlled session, and generation of a large cohort of embryos [2]. ICSI allows embryologists to select morphologically normal appearing sperm before injecting it directly into each oocyte for fertilization. Traditionally, sperm and oocytes are placed separately onto the ICSI dish, the spermatozoa are selected based on the morphologic characteristics and motility, and oocytes are loaded under the microscope and injected with a single spermatozoon one at a time. This method, despite its wide use, presents inherent limitations in methodology. Most notably, during sperm selection, delicate oocytes are exposed to a harsh microenvironment outside the incubator under the microscope while waiting for sperm injection, which can lead to oocyte damage. Numerous variables such as temperature fluctuations outside the incubator can harm both sperm and oocytes [3]. Studies have also shown that without light filters, particular wavelengths can damage gamete membranes [4,5]. Oocyte exposure may be particularly prolonged in cases of severe male factor infertility, defined as patients with fewer than 5 million motile sperm, where selection is prolonged due to decreased number and quality of the sperm [6]. Oocytes that have prior impaired quality may be at higher risk of damage under suboptimal conditions [7]. It has even been proposed that the epigenetic damage that gametes sustain during the ICSI process may have lifelong health consequences as a result of stress-related alterations in DNA methylation patterns [8]. By optimizing the efficiency of the proposed stepwise method of ICSI, fertility outcomes may be improved.

We aim to expand on a more efficient ICSI approach, named the pre-catching sperm (PCS) technique, as a method to reduce oocyte exposure time by selecting high-quality sperm before the oocyte is removed from the incubator. With this study, our objective is to measure the oocyte exposure times and fertilization and embryo development outcomes using the conventional and PCS technique. Our primary outcome measures are rate of fertilization, rate of oocyte degeneration, and rate of good quality blastocyst formation. Our secondary outcome is positive pregnancy rate. We hypothesize that with PCS technique we would observe better fertilization rates, lower degeneration rates, higher quality embryo formation rates, and higher positive pregnancy rates.

## 2. Materials and Methods

A retrospective cohort study was conducted comparing conventional ICSI with a PCS-ICSI technique between 2020 and 2023 at University Hospitals Fertility center. The stimulation protocol in both groups is in Table 1. Cases with at least 0.5 million total motile sperm and at least 5 mature oocytes were included. Between 3–5 h post-oocyte retrieval, the ICSI process was initiated as per standard practice by fully trained two embryologists. During the ICSI procedure, Ovoil Heavy (Vitrolife, Gothenburg, Sweden) was used as an overall plate medium to prevent desiccation of the gametes. Sperm were placed in 7% polyvinylpyrrolidone (PVP) droplets (Irvine Scientific, Santa Anna, CA, USA), and oocytes were placed in G-MOPS PLUS (Vitrolife, Gothenburg, Sweden) medium.

**Figure 1:**
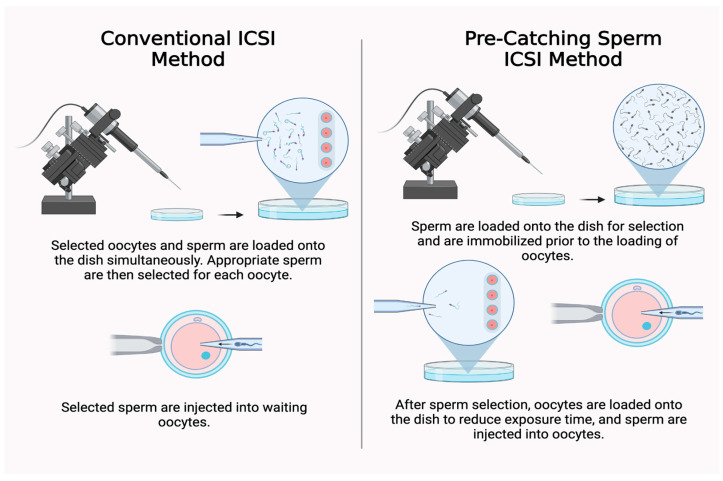
Conventional ICSI method (**left**) and PCS-ICSI method (**right**) (BioRender Software Version 2.0).

In the conventional ICSI technique, sperm and oocytes were simultaneously loaded onto the ICSI dish and up to four sperm were identified, immobilized, washed, and loaded into the pipette to be injected into the waiting oocytes (Figure 1). This process was repeated in groups of four until all oocytes in the cohort were fertilized. In the PCS-ICSI technique, the sperm was first loaded onto the ICSI dish in a 7% PVP drop and suitable sperm were identified, washed, and immobilized prior to the loading of the oocytes. Oocytes were then loaded onto the dish into G-MOPS PLUS medium and pre-caught sperm were injected into each oocyte as is done in the conventional ICSI method (Figure 1). In both methods, the oocytes were positioned so that the polar body is at the 6 or 12 o’clock position and the injection pipette was pushed to the tip of the oolemma against the zona to determine if the proper plane and positioning was accurate. The sperm was brought down via micromanipulation to the tip of the injection pipette and the tip was carefully inserted into the oocyte. The ooplasm was then aspirated until the plasma membrane was broken and the sperm was dispelled into the oocyte along with the aspirated ooplasm. All remaining oocytes were injected in the same manner with prepared and immobilized sperm. All injected oocytes were placed in a culture dish for fertilization assessment 16–20 h post injection per routine protocol.

The demographic variables were collected from female patient chart review: age, BMI, anti-Müllerian hormone level, baseline estradiol (E2), and peak estradiol (E2) (Table 2). The variables of interest included the oocyte fertilization and degeneration rates, abnormal fertilization, good quality blastocyst formation, number of frozen blastocysts, and pregnancy outcomes. Fertilized oocytes were assessed for degeneration immediately after the ICSI procedure as well as 16–20 h post-injection; the markers of degeneration included brown hue and grainy cytoplasm. Good-quality blastocysts were assessed using the Gardner embryo grading system, based on the degree of expansion and growth of the inner cell mass and trophectoderm [9]. Embryos that were grade 3BB or higher were considered to be good-quality embryos suitable for eventual implantation. Pregnancy rate was defined as beta-HCG level over 25 mIU/mL from medical record review, and clinical pregnancy was defined as presence of a gestational sac or fetal heartbeat on ultrasound from medical record review.

Student’s *t*-test and Fisher’s Exact Test were used for statistical analysis with *p* ≤ 0.05 considered statistically significant. Graph Pad Prism (version 10) was used for statistical analysis. The Shapiro–Wilk test was used to test for normality and the data were found to be normally distributed. The median with interquartile range (IQR) was used to show data distribution. The Median was chosen to show the whole number when possible.

## 3. Results

Our study included a total of 617 ICSI cases, with 330 cases using the PCS-ICSI technique and 287 control cases using the conventional ICSI technique. There was no significant difference in the total number of collected oocytes between the PCS and conventional groups (3840 vs. 3387, *p* = ns) (Table 2). The baseline characteristics, such as female age (35 years in the PCS group vs. 35 years in the control group, *p* = ns), body mass index (BMI; 27 vs. 26 *p* = ns), and anti-Müllerian hormone (AMH; 2.7 ng/mL vs. 3.0 ng/mL *p* = ns), were comparable between the two groups with no statistically significant differences. Baseline estradiol levels (24 pg/mL vs. 15 pg/mL; *p* = 0.002) and peak estradiol (2766 pg/mL vs. 2414 pg/mL; *p* = 0.01) were greater in the PCS group. Student’s *t*-test and Fisher’s Exact Test were used for statistical analysis with *p* ≤ 0.05 considered statistically significant.

The fertilization outcomes demonstrated a significantly higher oocyte fertilization rate in the PCS-ICSI group compared to the conventional group (84.0% vs. 79.3%, *p* < 0.001) (Table 3). The PCS group also exhibited a notably lower rate of oocyte degeneration compared to the control group (1.4% vs. 3.5%, *p* < 0.001), meaning that the reduced exposure time by approximately 5 min and optimized handling during the PCS technique may minimize oocyte damage.

The rate of abnormal fertilization was similar between the PCS and conventional groups (1.4% vs. 1.8%, *p* = 0.1601), suggesting that the PCS method does not increase the risk of fertilization anomalies (Table 3). When evaluating the quality of embryos, the PCS-ICSI technique resulted in a significantly higher formation rate of good-quality blastocysts (54.9% vs. 48.0%, *p* < 0.001). This finding indicates that the PCS method may promote better embryo development and enhance the potential for successful implantation.

A total of 1050 embryo transfer cases were reviewed for clinical pregnancy outcomes, including 434 embryo transfer cases from the PCS fertilization technique and 616 embryo transfer cases from the conventional fertilization technique. The positive pregnancy, clinical pregnancy and livebirth rates were similar among the groups (Table 4). Student’s *t*-test and Fisher’s Exact Test were used for statistical analysis with *p* ≤ 0.05 considered statistically significant.

## 4. Discussion

The results of our study demonstrate that the pre-catching sperm (PCS)-ICSI technique significantly improves several key metrics in the assisted reproductive process compared to the conventional ICSI method. Specifically, the PCS method led to a higher rate of oocyte fertilization and a lower rate of oocyte degeneration. Previous studies have shown that oocyte degeneration rates using the traditional technique are around 5–10% and can be as high as 20% [7,10]. Our PCS method shows promise, with an oocyte degeneration rate of only 1.4% vs. 3.5% in the conventional group, demonstrating both strength in our overall operator skill and efficient PCS methodology. This outcome has important implications for both male and female factor infertility that could not be managed with in vitro or intrauterine insemination [11]. Severe male factor infertility, defined as patients with fewer than 5 million motile sperm, often leads to prolonged sperm selection due to difficulty in finding suitable spermatozoa, resulting in longer oocyte exposure times under the microscope [6]. Female factor infertility, including abnormalities in zona pellucida, overall oocyte morphology, or limited quantity of oocytes, would also suffer damage from prolonged exposure times to already delicate oocytes [12]. With a degeneration rate of 1.4% in the PCS-ICSI group, our study findings suggest that reducing oocyte exposure time and optimizing the sperm selection process can minimize oocyte damage and enhance fertilization efficiency. To our knowledge, this is the first study qualifying fertility outcomes between distinct stepwise methodologies.

The PCS technique resulted in a significantly higher rate of good-quality blastocyst formation. Blastocyst quality is based on the of degree of cavity expansion, appearance of inner cell mass, and appearance of the trophectoderm [13]. Despite these advantages, the clinical pregnancy and ongoing/live birth rates were similar between the PCS and conventional ICSI groups. This indicates that while the PCS method enhances early stages of embryo development, it does not significantly impact long-term pregnancy outcomes when measured per transfer. This can also be attributed to the multifactorial nature of the obstetric course from implantation to delivery. This outcome also aligns with several studies. A 2017 study by Liu et al. found that there was no difference in implantation or pregnancy rates between a group that had at least one degenerated oocyte after ICSI and a group that had no degenerated oocytes after ICSI [14]. Another study by Hu et al. agreed that oocyte degeneration rate has no significant effect on pregnancy outcomes [10]. Similarly, our study results suggest that the rate of good-quality blastocyst formation also does not have a significant effect on the rate of pregnancy outcomes. However, the higher number of frozen blastocysts obtained through the PCS technique may have important implications for cumulative live birth rates. A greater number of good-quality frozen embryos increase the potential for successful outcomes in subsequent embryo transfer cycles. This potentially lowers the rate of patients needing to undergo repeat ovarian stimulation and oocyte retrieval procedures and improves the live birth rate per autologous retrieval regardless of its measured effect on pregnancy outcomes. This aspect of the PCS technique could offer significant benefits for patients undergoing assisted reproduction, as it provides more opportunities for achieving a successful pregnancy without the need for additional costly oocyte retrieval procedures [2]. Finally, due to requiring advanced technical skills, senior embryologists tend to complete ICSI procedures more quickly than junior embryologists. Most of the procedural time under the microscope is spent on sperm selection and isolation, which may be more challenging for junior embryologists to complete efficiently. The PCS technique could be particularly helpful for junior embryologists, as it allows them to separate the sperm selection and injection steps during ICSI procedure.

In addition to our PCS method, there is growing interest in other innovative ICSI techniques that have shown recent promise. The most technically difficult and error-prone step of ICSI is the manual injection of sperm into the oocyte and is the step that can directly lead to oocyte degeneration [15]. The piezo-ICSI technique overcomes this by using high-speed piezoelectric pulses of a micropipette to facilitate the injection of sperm into the oocyte. Piezo-ICSI assists with rapid, controlled penetration of the zona pellucida, and has been reported to reduce mechanical stress on the oocyte and improve fertilization rates [15,16,17]. Studies comparing piezo-ICSI with conventional ICSI have shown promising results, with higher fertilization rates, better embryo quality, and lower rates of degeneration [15,16,17]. This method has also been shown to improve ICSI success rates independently of operator experience level, thus increasing safety and improving outcomes when less experienced embryologists are performing ICSI [18]. Similarly to our study results, the piezo-ICSI technique does not appear to affect clinical pregnancy rates or live birth outcomes in smaller cohorts [15,16,17]. However, there is relatively little data on pregnancy outcomes using the piezo-ICSI technique in larger cohorts. Some studies suggest that patients seeking ICSI may also have additive fertility limitations that affect clinical pregnancy even after successful implantation and blastocyst formation [19]. Regardless, the piezo method could potentially complement or even enhance the improvements observed with the PCS technique and should be explored as a future area of research. Because the success of ICSI largely depends on the individual embryologist’s skill and competency, randomized controlled trials with larger, multi-center sample sizes are needed to ensure more generalizable and reliable results of PCS technique. 

This article is a revised and expanded version of a paper entitled Efficiency in Intracytoplasmic Sperm Injection Method Improves Fertilization and Blastocyst Formation Rates, which was presented at The Annual Congress of Pacific Coast Reproductive Society, Indian Wells, CA, USA, 20–23 March 2024 [20].

## 5. Conclusions

Our modification of the ICSI technique with increased efficiency resulted in an increased rate of oocyte fertilization and a higher yield of good quality blastocyst formation. Despite similar ongoing/live birth rates between the PCS and conventional ICSI groups, the higher number of frozen embryos obtained with the PCS technique may lead to higher cumulative live birth rates per autologous retrieval. To our knowledge, this is the only study comparing two stepwise ICSI methodologies on a broad range of outcomes. Future research should continue to explore the potential benefits of combining the PCS technique with emerging methods like piezo-ICSI to further improve outcomes in assisted reproductive technologies.

## Figures and Tables

**Table 1:** IVF Stimulation Protocol.

Stimulation Protocol	PCS (n = 330)	Conventional (n = 287)
Agonist Flare	52 (15.8%)	45 (15.7%)
Agonist suppression	40 (12.1%)	25 (8.7%)
Antagonist suppression	238 (72.1%)	217 (75.6%)

**Table 2:** ICSI demographic data comparing PCS and conventional methods.

Demographic Variables	PCS (n = 330)	Conventional (n = 287)	*p*-Value
Age (yr)	35 [32, 38]	35 [32, 38]	ns *
BMI (kg/m^2^) *	27 [23, 33]	26 [22, 30]	ns *
AMH (ng/mL) *	2.7 [1.6, 4.9]	3.0 [1.8, 4.6]	ns *
Baseline E2 (pg/mL) *	24 [20, 43]	15 [0, 29]	0.002
Peak E2 (pg/mL) *	2766 [1975, 3897]	2414 [1703, 3397]	<0.001

* BMI body mass index, AMH anti-Müllerian hormone, E2 estradiol, ns non-significant.

**Table 3:** ICSI outcomes data comparing PCS and conventional methods.

	PCS (n = 330)	Conventional (n = 287)	*p*-Value
**No. of retrieved mature oocytes**	3840	3387	ns *
**Fertilized oocytes (%)**	84.0	79.3	<0.001
**Degenerated oocytes (%)**	1.4	3.5	<0.001
**Abnormal fertilization (%)**	1.4	1.8	0.1601
**Good-quality blastocyst (%)**	54.9	48.0	<0.001

* ns non-significant.

**Table 4:** Pregnancy demographic and outcomes data per embryo transfer comparing PCS and conventional methods.

	PCS (n = 434)	Conventional (n = 616)	*p*-Value
Average age	35.4	35.6	ns *
Avg. number transferred embryo	1.1	1.2	ns *
Pregnancy rate (%)	78.8	75.8	0.2646
Clinical pregnancy (%)	69.6	66.9	0.3826
Ongoing/live birth (%)	57.6	55.7	0.5695

* ns non-significant.

## Data Availability

The raw data supporting the conclusions of this article will be made available by the authors on request.
